# 719 Effects of Obesity on Outcomes of Adult Burn Patients at a Single Institution

**DOI:** 10.1093/jbcr/irac012.273

**Published:** 2022-03-23

**Authors:** Sanja Sljivic, Lori Chrisco, Rabia Nizamani, Booker King, Felicia Williams

**Affiliations:** University of North Carolina - Chapel Hill, Chapel Hill, North Carolina; North Carolina Jaycee Burn Center, Chapel Hill, North Carolina; UNC Jaycee Burn Center, Chapel Hill, North Carolina; UNC Jaycee Burn Center, Chapel Hill, North Carolina; UNC Jaycee Burn Center, Chapel Hill, North Carolina

## Abstract

**Introduction:**

Obesity is a global epidemic that continues to worsen. In 2016, more than 1.9 billion adults were considered overweight worldwide and over 650 million were obese. It is well-known that excess adipose tissue may alter inflammatory and immune mediator regulations, which can lead to challenges in managing resuscitation efforts, respiratory support, and thromboprophylaxis of burn patients. The objective of this study was to evaluate the outcomes of burn patients with obesity at our institution.

**Methods:**

This was a single-site, retrospective review using our institutional Burn Center registry. All adult patients (18 years or older) admitted to our Burn Center between July 1, 2013 and June 30, 2021 who were classified as obese (i.e., body mass index > 30.0) were included in this study. All adult patients who were classified as underweight, normal weight, or overweight were also included for comparative purposes. Variables of interest included demographics, burn mechanism, length of stay (LOS), cost of hospitalization, and mortality.

**Results:**

There were 7,626 patients included in this study, with the largest percentage of patients included in the obese category (38.4%). Among the obese population, most of the patients (53.2%) were classified under ‘Obesity Class I’ (i.e., body mass index 30.0 – 34.9). The majority of patients in each category were male, except in the ‘Obesity Class III’ category (i.e., body mass index > 40.0) where 54.8% of the population were female. The mean age of the entire study sample was 44.9 years +/- 17.5 years, while the mean total body surface area (TBSA) involvement was 5.1% +/- 10.0%. The mean LOS of the entire study population was 10.3 days +/- 22.6 days, with patients in the ‘Obesity Class III’ category having the longest LOS with 14.0 days +/- 36.5 days. The cost of hospitalization was lowest in the overweight group with $82,661, while the highest cost was in the ‘Obesity Class III’ group with $130,683. The overall hospital mortality for the entire study population was 3.0% with the highest mortality noted in the ‘Obesity Class III’ group (4.7%).

**Conclusions:**

Obesity affects all aspects of a burn patient’s care throughout their hospitalization. In our study, obesity was associated with longer LOS, cost of hospitalization, and mortality; therefore, it is imperative to understand the negative effects that obesity can have on burn patients, not just in terms of their acute management, but also their continued care after hospitalization.

